# Synthetic microbial community promotes seedling growth of Chinese fir via dissolving phosphorus and modifying rhizosphere microbial community

**DOI:** 10.1093/treephys/tpag135

**Published:** 2026-09-21

**Authors:** Junhao Zhang, Songxie Xu, Chaoying Chen, Zhanling Wang, Wenjun Du, Liang Hong, Fred O Asiegbu, Kai Wang

**Affiliations:** College of Forestry, Fujian Agriculture and Forestry University, No. 15 Shangxiadian Road, Cangshan District, Fuzhou 350002, China; College of Forestry, Fujian Agriculture and Forestry University, No. 15 Shangxiadian Road, Cangshan District, Fuzhou 350002, China; College of Forestry, Fujian Agriculture and Forestry University, No. 15 Shangxiadian Road, Cangshan District, Fuzhou 350002, China; College of Forestry, Fujian Agriculture and Forestry University, No. 15 Shangxiadian Road, Cangshan District, Fuzhou 350002, China; Chinese Fir Engineering Research Center of National Forestry and Grassland Administration, College of Forestry, Fujian Agriculture and Forestry University, No. 15 Shangxiadian Road, Cangshan District, Fuzhou 350002, China; College of Forestry, Fujian Agriculture and Forestry University, No. 15 Shangxiadian Road, Cangshan District, Fuzhou 350002, China; Chinese Fir Engineering Research Center of National Forestry and Grassland Administration, College of Forestry, Fujian Agriculture and Forestry University, No. 15 Shangxiadian Road, Cangshan District, Fuzhou 350002, China; College of Forestry, Fujian Agriculture and Forestry University, No. 15 Shangxiadian Road, Cangshan District, Fuzhou 350002, China; Chinese Fir Engineering Research Center of National Forestry and Grassland Administration, College of Forestry, Fujian Agriculture and Forestry University, No. 15 Shangxiadian Road, Cangshan District, Fuzhou 350002, China; Faculty of Agriculture and Forestry, University of Helsinki, FIN-00014 Helsinki, PO Box 27, Finland; College of Forestry, Fujian Agriculture and Forestry University, No. 15 Shangxiadian Road, Cangshan District, Fuzhou 350002, China; Chinese Fir Engineering Research Center of National Forestry and Grassland Administration, College of Forestry, Fujian Agriculture and Forestry University, No. 15 Shangxiadian Road, Cangshan District, Fuzhou 350002, China; Faculty of Agriculture and Forestry, University of Helsinki, FIN-00014 Helsinki, PO Box 27, Finland

**Keywords:** bacterial bioinoculant, *Cunninghamia lanceolata*, endophytic bacteria, metagenome, phosphate solubilization

## Abstract

Phosphorus (P) is an essential nutrient for plant growth, yet its availability in soil is severely constrained by fixation into insoluble forms that plants cannot directly utilize. Although phosphate-solubilizing microorganisms (PSM) represent a promising strategy to mobilize soil phosphorus, the functional potential of endophytic PSM and their synthetic consortia in promoting tree growth remains largely underexplored. In this study, endophytic bacteria were isolated from the roots of Chinese fir (*Cunninghamia lanceolata* (Lamb.) Hook.). Among them, 42 isolates were screened for phosphate-solubilizing activity on media containing calcium phosphate, iron phosphate, aluminum phosphate and organic phosphorus. Six strains with strong solubilizing capacity and no antagonistic interactions were selected to construct a synthetic microbial community (SynCom-P6), which was then applied to local soil and Chinese fir seedlings. Soil available phosphorus content initially decreased but subsequently increased from day 7 to day 21 after SynCom-P6 inoculation, indicating effective mobilization of insoluble phosphorus in soil. Inoculation with SynCom-P6 significantly enhanced root elongation and biomass accumulation, with both root fresh and dry weights showing marked increases compared with the control. Absolute quantification 16S amplicon and metagenomic sequencing revealed that SynCom-P6 reshaped the rhizosphere bacterial community, enriching beneficial genera such as *Massilia* and *Dyadobacter*, and altered functional profiles, including upregulation of hormone signaling and nitrogen fixation–related genes. These results demonstrated that the synthetic community not only improved soil phosphorus availability but also promoted root growth and modified the rhizosphere microbiome in a beneficial direction. Our findings highlight the potential of endophytic phosphate-solubilizing SynCom-P6 as a promising bio-inoculant for sustainable forestry, reducing the need for chemical P fertilizers while enhancing Chinese fir productivity. Future research should focus on field validation and mechanistic exploration of microbial interactions and functional gene expression.

## Introduction

Phosphorus is one of the most important nutrient elements essential for plant growth and development (McDowell et al. 2024). It is a limited resource in soil and is continuously decreasing (Sattari et al. 2012). Among the nutrient elements, the effect of phosphorus on the productivity and health of terrestrial and aquatic ecosystems is second only to nitrogen (Brady and Weil 2002). Compared with other essential mineral elements for plants, phosphorus has low availability and poor mobility in soil, resulting in insufficient available phosphorus supply in some soil types (Li et al. 2017). In Northern China, calcium phosphate is the dominant form of phosphorus due to the dry and cold climate and relatively poor soil conditions. Unlike Northern China, the humid climate and high organic matter content in Southern China make aluminum phosphate and iron phosphate the main phosphorus forms in soil. Ultimately, most soil phosphorus in both northern and southern regions is tightly fixed on soil surface particles or bound (immobilized) in the form of organophosphorus compounds and cannot be absorbed by plants (Kochian 2012), leading to a decrease in available phosphorus content in soil. People have realized that improving plant utilization efficiency of soil phosphorus can increase yield (Syers et al. 2008), so phosphate fertilizer application has been adopted widely. However, most soluble phosphorus applied to soil via fertilizers is also rapidly immobilized through specific adsorption or chemical precipitation, and transformed into non-available phosphorus that is difficult for plants to absorb and utilize.

There are abundant insoluble phosphorus salts in soil that are hardly directly absorbable and utilizable by plants. Phosphorus deficiency in plants causes deficiency symptoms, thereby restricting the growth of Chinese fir (*Cunninghamia lanceolata* (Lamb.) Hook.), a highly economically important tree species in Asia. Therefore, phosphorus deficiency is widespread in many natural ecosystems, including forestry plantations (Fang et al. 2024). As plant yields increase, phosphorus fertilizer usage continues to rise. However, the seasonal utilization rate of phosphorus fertilizers by crops typically ranges only between 5% and 10%. Even when accounting for aftereffects of crops, the total utilization rate does not exceed 25% (Lu 2003). Supplementing phosphorus required by Chinese fir through fertilization will not only increase the content of potentially harmful trace elements such as arsenic, cadmium and lead in soil (Jiao et al. 2012), but also cause phosphorus loading, leading to excessive growth of phytoplankton or macroalgae in the region and stimulating the growth of aquatic bacteria and fecal bacteria, thus causing environmental damage (Mallin and Cahoon 2020). Therefore, improving the absorption and utilization of phosphorus by crops is of great significance from both ecological and economic perspectives (Abbas et al. 2018).

Soil microbiomes drive key functions in agricultural and forestry ecosystems, determining characteristics such as soil fertility, plant productivity, stress resistance and health status (Hartmann and Six 2023, Hong et al. 2024, Wang et al. 2026). The limitation of plant phosphorus uptake from soil is mainly due to phosphorus fixation on soil particles. To increase soil available phosphorus content, scientists have identified soil microorganisms belonging to specific functional groups, which are generally referred to as phosphate-solubilizing microorganisms (Khan et al. 2013). These are a group of microorganisms that can solubilize insoluble phosphates, fix nitrogen and secrete auxin to promote plant growth (Chen et al. 2021, Wang et al. 2026). The phosphorus fixed in the soil is dissolved by phosphate-solubilizing microorganisms. These bacteria release different types of organic acids in the soil that can convert insoluble phosphorus into soluble phosphorus for plant absorption and utilization. Plants enhance phosphorus absorption by associating with microorganisms that promote phosphorus acquisition, thereby improving plant utilization efficiency of soil phosphorus (Teng et al. 2017, Billah et al. 2019, Oldroyd and Leyser 2020). These phosphate-solubilizing bacteria can not only supply phosphorus to plants but also promote plant growth (Zaidi et al. 2009). Three phosphate-solubilizing bacteria, *Pantoea ananatis*, *Rahnella aquatilis* and *Enterobacter* sp., can release potassium from mica and promote rice plant growth at an early stage of development (Bakhshandeh et al. 2017). Inoculation with phosphate-solubilizing bacteria can increase seed cotton yield, boll number per plant and boll weight (Qureshi et al. 2012). Two phosphate-solubilizing strains EB 67 and CDB 35 can increase maize yield by 85% and 64%, respectively (Hameeda et al. 2008). Increasing soil available phosphorus content by applying phosphate-solubilizing bacteria can reduce environmental pollution caused by fertilizer application and play an important role in promoting ecosystem balance (Zak et al. 2018).

Endophytes are microbes that live in the interior of plants at a certain or at all stages (Hardoim et al. 2008). As an important component of plant holobionts, they are ubiquitous in most higher plants (Lodewyckx et al. 2002). Endophytes can promote plant growth, elicit defense responses against pathogen infection, and act as ameliorating agents under abiotic stress (Khare et al. 2018, Hong et al. 2024). Plant growth–promoting bacteria that fix nitrogen, solubilize phosphorus and mobilize potassium to enhance plant growth (Lu et al. 2000), biocontrol bacteria for forest protection (Faissal et al. 2017) and beneficial bacteria that improve plant stress resistance have been identified (Li et al. 2012). Synthetic microbial communities (SynComs) are artificially assembled from two or more cultivated microbial strains (Li et al. 2024). SynComs can be constructed by selecting key members from natural microbial communities or by considering predicted microbial interactions. These carefully designed communities can reshape the structure and function of the plant microbiome, improve soil fertility and plant performance, and provide a novel strategy for regulating plant-associated microbial communities and enhancing crop protection (Chaudhary et al. 2023, Sarver et al. 2025). SynComs enable controlled investigations of community functions by reducing the complexity of natural microbial communities while retaining their core functions (Großkopf and Soyer 2014). SynComs constructed with phosphate-related bacterial strains have been shown to enhance phosphorus uptake and root growth in rice (Ma et al. 2024). At present, SynComs were increasingly used to explore plant-microbe interactions and nutrient cycling.

Chinese fir is the largest timber tree species in Southern China (Wu et al. 2019). It has high economic and ecological value and is also an important supporting tree species for the construction of commercial forest bases in Southern China. However, due to the acidic, barren and sticky characteristics of the red soil, coupled with the uneven spatial and temporal precipitation in the distribution area and the management methods of continuous rotation and clear cutting, the yield of Chinese fir decreased (Zhao et al. 2013, Farooq et al. 2019). Therefore, it is of great practical significance to study endophytic bacteria with phosphate-solubilizing ability in Chinese fir roots, isolate and purify these strains, optimize their culture conditions, and apply them to Chinese fir and soil to efficiently convert insoluble phosphorus into available phosphorus, improve soil fertility and promote the growth of Chinese fir (Wang et al. 2026).

Our previous work showed that fine roots of Chinese fir harbor endophytic bacteria with phosphate-solubilizing potential (Wang et al. 2024). However, whether these endophyte-derived phosphate-solubilizing bacteria can be rationally assembled into a synthetic microbial community and whether such a community can improve soil phosphorus availability, seedling growth and rhizosphere microbial functions in Chinese fir remain unclear.

In this study, we tested the following hypotheses:

H1: endophytic bacteria isolated from Chinese fir roots differ in their ability to solubilize distinct inorganic and organic phosphorus sources, allowing the selection of functionally active phosphate-solubilizing strains for SynCom-P6 construction.

H2: a synthetic microbial community assembled from compatible phosphate-solubilizing endophytes increases soil available phosphorus in a controlled soil culture experiment.

H3: inoculation with this SynCom-P6 promotes the growth of in-vitro Chinese fir seedlings, particularly root elongation and belowground biomass accumulation.

H4: SynCom-P6 inoculation reshapes the rhizosphere microbial community and shifts metagenomic functional profiles associated with nutrient cycling and plant growth promotion.

To test these hypotheses, we first screened 42 endophytic bacterial isolates from Chinese fir roots using four phosphate-containing media. Six isolates with strong phosphate-solubilizing activity and no detectable antagonism were selected to construct a synthetic microbial community, hereafter referred to as SynCom-P6. We then evaluated SynCom-P6 in two controlled experiments: a soil microcosm experiment designed to quantify temporal changes in soil available phosphorus, and an in-vitro seedling experiment designed to assess seedling growth responses. Finally, absolute-quantification 16S rRNA amplicon sequencing and metagenomic sequencing were used to determine whether SynCom-P6 altered rhizosphere microbial community structure and functional potential. This design allowed us to link strain-level phosphate-solubilizing capacity, soil phosphorus dynamics, plant growth responses, and microbiome-level changes in Chinese fir.

## Materials and methods

### Experimental design

This study mainly included three parts: screening and construction of the synthetic microbial community, soil culture experiment, and inoculation experiment of Chinese fir test-tube seedlings. The purpose of this study was to evaluate whether the synthetic community constructed from endophytic phosphate-solubilizing bacteria could increase soil available phosphorus, promote the growth of Chinese fir seedlings, and change the rhizosphere microbial community.

Firstly, 42 endophytic bacterial strains isolated from Chinese fir roots were screened on four phosphate-containing media, including calcium phosphate, iron phosphate, aluminum phosphate, and Mongina organic phosphorus medium. The strains with obvious phosphate-solubilizing halos were recorded. Six strains with strong phosphate-solubilizing ability were selected to construct the synthetic microbial community. Before construction, pairwise antagonism tests were carried out among the six strains on LB medium. Strains without obvious antagonistic effects were used for the final synthetic community, named SynCom-P6.

Secondly, the soil culture experiment was carried out to test the effect of SynCom-P6 on soil available phosphorus. The experiment included two treatments: control and SynCom-P6 inoculation. The control group was inoculated with sterile 10 mM MgCl₂ solution, and the treatment group was inoculated with SynCom-P6 suspension. Soil samples were collected at four time points: day 0, day 7, day 14 and day 21 after inoculation. Each treatment at each time point contained three biological replicates. Therefore, a total of 24 sterile 50-mL tubes were prepared, and each tube contained 30 g of pre-treated soil. Each control tube received 5 mL of sterile 10 mM MgCl₂ solution, while each SynCom-P6 tube received 5 mL of the synthetic community suspension. Soil available phosphorus was measured for each biological replicate.

Thirdly, the inoculation experiment of Chinese fir test-tube seedlings was carried out to evaluate the growth-promoting effect of SynCom-P6. Chinese fir plantlets grown in vitro with similar growth status were selected and randomly divided into control and SynCom-P6 treatment groups. Each treatment contained 20 independent seedlings. The control seedlings were inoculated with 5 mL of sterile 10 mM MgCl₂ solution, while the SynCom-P6 seedlings were inoculated with 5 mL of SynCom-P6 suspension adjusted to OD_600_ = 0.2. After inoculation, all seedlings were maintained in a growth chamber for 5 months. At the end of the experiment, seedling height, root length, shoot fresh weight, shoot dry weight, root fresh weight and root dry weight were measured.

Rhizosphere soil samples were collected from the seedlings at the end of the inoculation experiment for microbial community analysis. For each treatment, rhizosphere soil from 20 seedlings was divided into five pooled biological replicates, with each pooled replicate consisting of rhizosphere soil collected from four independent seedlings. The same five pooled biological replicates per treatment were used for absolute-quantification 16S rRNA amplicon sequencing and metagenomic sequencing. These samples were used for bacterial community composition, alpha diversity, beta diversity, differential abundance and metagenomic functional annotation analyses.

### Screening of phosphate-solubilizing endophytic bacteria

Milli-Q water was added to a conical flask to prepare phosphate-containing media. Calcium phosphate, iron phosphate, aluminum phosphate and Mongina organic phosphorus medium were prepared according to their respective formulations. The required reagents were accurately weighed and transferred into the flask. The suspension was heated in a water bath at 70 °C for 15 min with continuous stirring to facilitate dissolution. After complete dissolution, the pH was adjusted to 7.0 using a calibrated pH meter. The medium was transferred to autoclavable containers and sterilized at 121 °C for 15 min. After sterilization, the medium was gently mixed, cooled to ~50 °C, and aseptically dispensed into sterile Petri dishes with 10 mL medium per plate in a laminar flow hood. The plates were left to solidify at room temperature.

A sterile 10 mM MgCl₂ solution was prepared by dissolving 0.94 g MgCl₂ in 1 L of ultrapure water, followed by filtration through a 0.22 μm sterile microporous membrane under aseptic conditions. For bacterial inoculation, 600 μL of sterile 10 mM MgCl₂ solution was added into sterile microcentrifuge tubes. Each of the 42 endophytic bacterial strains isolated from Chinese fir roots was separately suspended in MgCl₂ solution and vortexed to obtain a uniform suspension. Then, 10 μL of each bacterial suspension was spotted onto the center of the pre-solidified phosphate-containing agar plate. After the inoculum was absorbed, the plates were sealed with sterile sealing film, labeled and incubated upside down at 28 °C.

Colony growth and halo formation were recorded during incubation. Strains showing transparent halos around colonies against the opaque background of insoluble phosphate were considered to have phosphate-solubilizing ability. The phosphate-solubilizing performance of each strain was evaluated according to colony growth and halo formation on the four phosphate-containing media.

Six isolates with strong phosphate-solubilizing activity were selected for taxonomic identification. Genomic DNA was extracted from pure cultures using the TIANamp Bacteria DNA Kit (TIANGEN, Beijing, China). The full-length 16S rRNA gene was amplified using primers 27F and 1492R, and the PCR products were subjected to Sanger sequencing by Sangon Biotech (Shanghai, China). The resulting sequences were compared with reference 16S rRNA gene sequences in the NCBI GenBank database using BLASTn for taxonomic assignment.

### Antagonism assay and construction of SynCom-P6

Six strains with stronger phosphate-solubilizing ability were selected for construction of the synthetic microbial community. Before mixing the strains, pairwise antagonism tests were carried out among the six strains on LB agar plates. For each pairwise combination, two strains were inoculated onto the same plate, and the two inoculation points were placed approximately one-quarter of the plate distance apart. Three biological replicates were prepared for each combination, and self-inoculation controls were set for each strain. The plates were incubated at 28 °C for 5 days. Antagonism was evaluated according to the presence of inhibition zones or abnormal colony growth between paired strains. No obvious antagonistic effect was observed among the six strains; therefore, all six strains were used to construct the final synthetic microbial community, named SynCom-P6.

LB medium was prepared by dissolving peptone or proteose peptone, yeast extract and NaCl in distilled water at final amounts of 10.0 g, 5.0 g and 10.0 g per liter, respectively. The pH was adjusted to 7.0 using a calibrated pH meter, and the medium was sterilized at 121 °C for 15 min. After sterilization, the medium was gently mixed and cooled to room temperature. A sterile 10 mM MgCl₂ solution was prepared as described above and used for bacterial washing, resuspension, and control inoculation.

Each of the six selected strains was cultured separately in liquid LB medium. Briefly, 30 mL aliquots of liquid LB medium were added into sterile 50-mL centrifuge tubes. The tubes were inoculated with the corresponding bacterial suspension and incubated at 28 °C with shaking at 200 r.p.m. for 20 h. Bacterial growth was confirmed by visible turbidity. After incubation, bacterial cells were collected by centrifugation at 5000 r.p.m. at 4 °C for 5 min. The supernatant was discarded, and the cell pellets were washed and resuspended in 15 mL sterile 10 mM MgCl₂ solution.

The optical density of each bacterial suspension was measured at 600 nm using a UV–Vis spectrophotometer. Each suspension was diluted with sterile 10 mM MgCl₂ solution to a final OD_600_ of 0.2 and adjusted to a final volume of 50 mL. Equal volumes of the six standardized bacterial suspensions were then mixed to obtain SynCom-P6. Sterile 10 mM MgCl₂ solution was used as the control inoculum in both the soil culture experiment and the Chinese fir seedling inoculation experiment.

### Application of SynCom-P6 to soil

The soil culture experiment was carried out to evaluate whether SynCom-P6 affected soil available phosphorus. A total of 24 sterile 50-mL tubes were prepared, and each tube contained 30 g of pre-treated test soil. The experiment included two treatments: control and SynCom-P6 inoculation. The control tubes were inoculated with 5 mL of sterile 10 mM MgCl₂ solution, while the SynCom-P6 tubes were inoculated with 5 mL of SynCom-P6 suspension.

Soil samples were collected at four time points: day 0, day 7, day 14 and day 21 after inoculation. For each treatment, three biological replicates were prepared at each sampling time. After inoculation, the soil and inoculum were mixed thoroughly, and all tubes were incubated at 28 °C. At each time point, the corresponding soil samples were collected, air-dried in a cool and ventilated place, and stored for the determination of soil available phosphorus.

### Measurement of soil available phosphorus

Air-dried soil samples were ground and passed through a 2-mm sieve. For each sample, 3.0 g of soil was weighed into a 50-mL plastic centrifuge tube. Then, 50 mL of Bray-1 extractant and a small amount of phosphorus-free activated carbon were added. The tubes were shaken at 200 r.p.m. for 30 min at 20–25 °C. After extraction, the suspension was filtered through phosphorus-free qualitative filter paper, and the filtrate was collected into 50-mL centrifuge tubes for colorimetric determination.

For color development, 10.0 mL of filtrate was transferred into a 50-mL volumetric flask. Then, 10 mL of boric acid solution and two drops of 2,4-dinitrophenol indicator were added. The pH was adjusted to a faint yellow color using 0.5 M HCl or 2 M NaOH. Subsequently, 5 mL of molybdenum-antimony tartrate color reagent was added, and the volume was adjusted to 50 mL with deionized water. After thorough mixing, the solution was allowed to develop color at room temperature above 15 °C for 30 min. Absorbance was measured at 700 nm using a UV–Vis spectrophotometer. A reagent blank, prepared using Bray-1 extractant and processed in the same way as the samples, was used as the reference.

A standard phosphorus series of 0, 0.1, 0.2, 0.3, 0.4, 0.5 and 0.6 mg P L^−1^ was prepared from a 100 mg P L^−1^ stock solution. The standard solutions were processed following the same colorimetric procedure as the samples. Soil available phosphorus concentration was calculated according to the linear regression equation of the standard curve. Soil available phosphorus content was calculated as follows:


$$ \omega \left(\mathrm{P}\right)=\left(\rho \times \mathrm{V}\times \mathrm{Ts}\times{10}^{-6}\right)\times 100/m $$

where ρ is the phosphorus concentration in the colorimetric solution calculated from the standard curve; V is the volume of the colorimetric solution; Ts is the dilution factor during sample extraction; 10^−6^ is the unit conversion coefficient; and m is the mass of the dried soil sample.

### Inoculation of SynCom-P6 to Chinese fir test-tube seedlings

Chinese fir plantlets grown in vitro were selected according to similar growth status and randomly divided into control and SynCom-P6 treatment groups. Each treatment contained 20 independent seedlings. The control seedlings were inoculated with 5 mL of sterile 10 mM MgCl₂ solution, while the SynCom-P6 seedlings were inoculated with 5 mL of SynCom-P6 suspension (OD_600_ = 0.2).

After inoculation, all seedlings were maintained in a growth chamber for five months under controlled temperature and photoperiod conditions. At the end of the experiment, seedling growth was recorded and photographed. Seedling height and root length were measured. Shoots and roots were separated, and fresh weight was measured immediately. The samples were then dried to constant weight to determine dry weight. The measured growth traits included seedling height, root length, shoot fresh weight, shoot dry weight, root fresh weight and root dry weight.

### Absolute-quantification 16S rRNA amplicon sequencing and analysis

Rhizosphere soil samples collected from the Chinese fir seedling inoculation experiment were used for absolute-quantification 16S rRNA amplicon sequencing. The control and SynCom-P6 treatments were both included. For each treatment, 20 seedlings were divided into five pooled biological replicates, and each pooled replicate consisted of rhizosphere soil collected from four independent seedlings. Therefore, five pooled biological replicates were used for each treatment.

Total genomic DNA was extracted from rhizosphere samples using a commercial DNA extraction kit. DNA integrity was checked by agarose gel electrophoresis, and clear bands without obvious degradation were regarded as qualified. DNA concentration was measured using Qubit, and all qualified samples were normalized to 10 ng μL^−1^, with each sample containing at least 500 ng of total DNA. Before formal library preparation, high-fidelity PCR pre-amplification of the target region was performed without spike-in DNA, together with a positive control consisting of standard bacterial genomic DNA mixture. Amplification products were checked by agarose gel electrophoresis, and qualified samples were used for subsequent library preparation.

During target-region amplification, sample DNA and spike-in DNA with known copy numbers were co-amplified in the same high-fidelity PCR system. Sample-specific index sequences were introduced during PCR using indexed primers. After index PCR, the products were checked by agarose gel electrophoresis. Libraries were diluted according to band intensity and quantified using Qubit. Qualified libraries were pooled in equimolar proportions according to their concentrations to meet the sequencing depth requirements. The pooled library was gel-purified to remove non-specific amplification products. Fragment size distribution was checked on an ABI 3730 system to confirm a peak size within the expected range. The Illumina-compatible library was then converted into a single-stranded circular DNA library using the MGI Easy Universal Library Conversion Kit for compatibility with the DNBSEQ platform. Final sequencing was performed on the DNBSEQ-G99RS platform.

The sequencing data were used to analyze bacterial community composition. Alpha diversity indices were calculated to evaluate bacterial richness and diversity. Bray–Curtis distance-based principal coordinate analysis was performed to compare bacterial community structure between the control and SynCom-P6 treatments. Differentially abundant genera were analyzed based on both relative abundance and absolute abundance.

### Absolute metagenomic sequencing and analysis

Rhizosphere soil samples collected from the Chinese fir seedling inoculation experiment were also used for absolute metagenomic sequencing. The control and SynCom-P6 treatments were both included. For each treatment, 20 seedlings were divided into five pooled biological replicates, and each pooled replicate consisted of rhizosphere soil collected from four independent seedlings. The same pooled replicate design was used for metagenomic sequencing as for 16S rRNA amplicon sequencing.

Metagenomic absolute quantification was performed using the AccuMetaG® protocol to characterize the functional potential of the rhizosphere microbial community. Total genomic DNA was extracted from rhizosphere samples using a commercial DNA extraction kit. DNA integrity was evaluated by agarose gel electrophoresis, and DNA concentration was measured using Qubit. Samples with high-quality DNA, with concentration ≥25 ng μL^−1^ and total yield ≥2 μg, were selected for library construction. Before library preparation, synthetic internal standard DNA with known copy numbers was added to each sample and mixed thoroughly to enable absolute quantification.

The DNA mixture was sheared into ~400-bp fragments using a Covaris ME220 focused ultrasonicator. Fragmented DNA was subjected to end repair, 3′-dA tailing, adapter ligation and limited-cycle PCR amplification using a high-fidelity polymerase. The amplified libraries were purified and screened according to concentration and fragment size using Qubit fluorometry and gel electrophoresis. Libraries with concentration >5 ng μL^−1^ and fragment size of 300–500 bp were regarded as qualified. Qualified libraries were sequenced on the DNBSEQ-T7 platform using a 2 × 150 bp paired-end sequencing strategy.

The raw metagenomic data were used for subsequent bioinformatic analysis. Functional annotation was carried out based on KEGG Orthology and UniProt databases. The proportions of mapped and unmapped sequences were calculated to evaluate the annotation results. Representative KEGG pathways were compared between the control and SynCom-P6 treatments. Nitrogen fixation–related genes were extracted from the annotated functional profiles and visualized using heatmaps.

### Statistical analyses

All statistical analyses were performed in R. Biological replicates were used as the units for analysis. In the soil culture experiment, each 50-mL tube represented one independent replicate. In the seedling inoculation experiment, each seedling was treated as one replicate for growth measurements. For 16S rRNA amplicon sequencing and metagenomic sequencing, each pooled rhizosphere sample was treated as one replicate. When repeated technical readings were obtained from the same replicate, their mean value was used for analysis.

For soil available phosphorus, reagent blanks were included throughout the colorimetric procedure and used for blank correction. Phosphorus concentrations were calculated from the standard curve, and the linearity of the curve was checked before sample calculation.

Before statistical testing, normality and homogeneity of variance were examined using the Shapiro–Wilk test and Levene’s test, respectively. For ANOVA models, residual plots were also checked. When necessary, log or square-root transformation was applied to improve the distribution of the data. Potential outliers were inspected using boxplots and standardized residuals. Data were excluded only when a clear technical or operational error could be identified; otherwise, all biological replicates were retained.

Soil available phosphorus was first analyzed by two-way ANOVA, with treatment, sampling time and their interaction as fixed factors. Because soils were destructively sampled at each time point, the tubes were considered independent rather than repeated measurements. When a significant treatment × time interaction was detected, comparisons between the control and SynCom-P6 treatments were made within each sampling time. Student’s *t-*test was used when the assumptions of normality and equal variance were met, and Welch’s *t-*test was used when variances were unequal. If the assumptions for parametric tests were still not met after transformation, the Wilcoxon rank-sum test was used. *P*-values from multiple pairwise comparisons were adjusted using the Benjamini–Hochberg false discovery rate method.

For the seedling inoculation experiment, seedling height, root length, shoot fresh weight, shoot dry weight, root fresh weight and root dry weight were compared between the control and SynCom-P6 treatments. Student’s *t-*test was used for normally distributed data with equal variance, Welch’s *t-*test for unequal variance and the Wilcoxon rank-sum test for data that did not meet parametric assumptions.

For the 16S rRNA amplicon data, alpha diversity indices, including Observed features, Chao1, ACE, Shannon, Simpson, Pielou’s evenness, Faith’s phylogenetic diversity and Good’s coverage, were compared between treatments using the Wilcoxon rank-sum test. Bray–Curtis dissimilarities were calculated from bacterial community profiles and visualized by principal coordinate analysis. Differences in community structure were tested by permutational multivariate analysis of variance with 999 permutations. Homogeneity of multivariate dispersion was checked before interpreting the PERMANOVA results. Genus-level differences in relative and absolute abundance were tested using the Wilcoxon rank-sum test, followed by Benjamini–Hochberg correction for multiple comparisons.

For the metagenomic data, Bray–Curtis dissimilarities were calculated from functional profiles and visualized by principal coordinate analysis. Treatment effects on functional composition were tested by PERMANOVA with 999 permutations, and homogeneity of multivariate dispersion was checked before interpretation. KEGG Orthology and UniProt annotation results were summarized to describe functional composition. Representative KEGG pathway abundances and functional genes were compared between treatments using the Wilcoxon rank-sum test, followed by Benjamini–Hochberg correction when multiple features were tested.

Nitrogen fixation-related genes were extracted from the annotated metagenomic profiles and shown in heatmaps. For visualization, gene abundances were transformed into row-wise Z-scores to show the relative enrichment pattern of each gene across samples. Z-scores were used only for visualization, whereas statistical tests were performed on abundance values before Z-score transformation. Unless otherwise stated, differences were considered significant at *P* < 0.05. Significance levels are indicated as ns, not significant; ^*^P < 0.05; ^**^P < 0.01; ^***^P < 0.001.

## Results

### Candidates of phosphate dissolving bacteria from Chinese fir

A total of 42 endophytic bacterial isolates were isolated and purified from Chinese fir roots. These isolates were individually inoculated onto three inorganic phosphate media (iron phosphate, aluminum phosphate and calcium phosphate) and one organic phosphate medium (Mongina), and then were incubated in a growth chamber. Phosphate-solubilizing halos were observed on day 3 and were documented by photography (Figure 1). Little to no halo formation was detected on the aluminum phosphate or iron phosphate media, whereas clear halos were observed on the Mongina and calcium phosphate media. In total, 15 isolates showed phosphate-solubilizing activity on the calcium phosphate and Mongina media. Among them, six isolates with stronger phosphate-solubilizing capacity (34, 35, 37, 40, 41 and 52) were identified by full-length 16S rRNA gene sequencing and selected for SynCom-P6 construction (Table 1).

**Figure 1:**
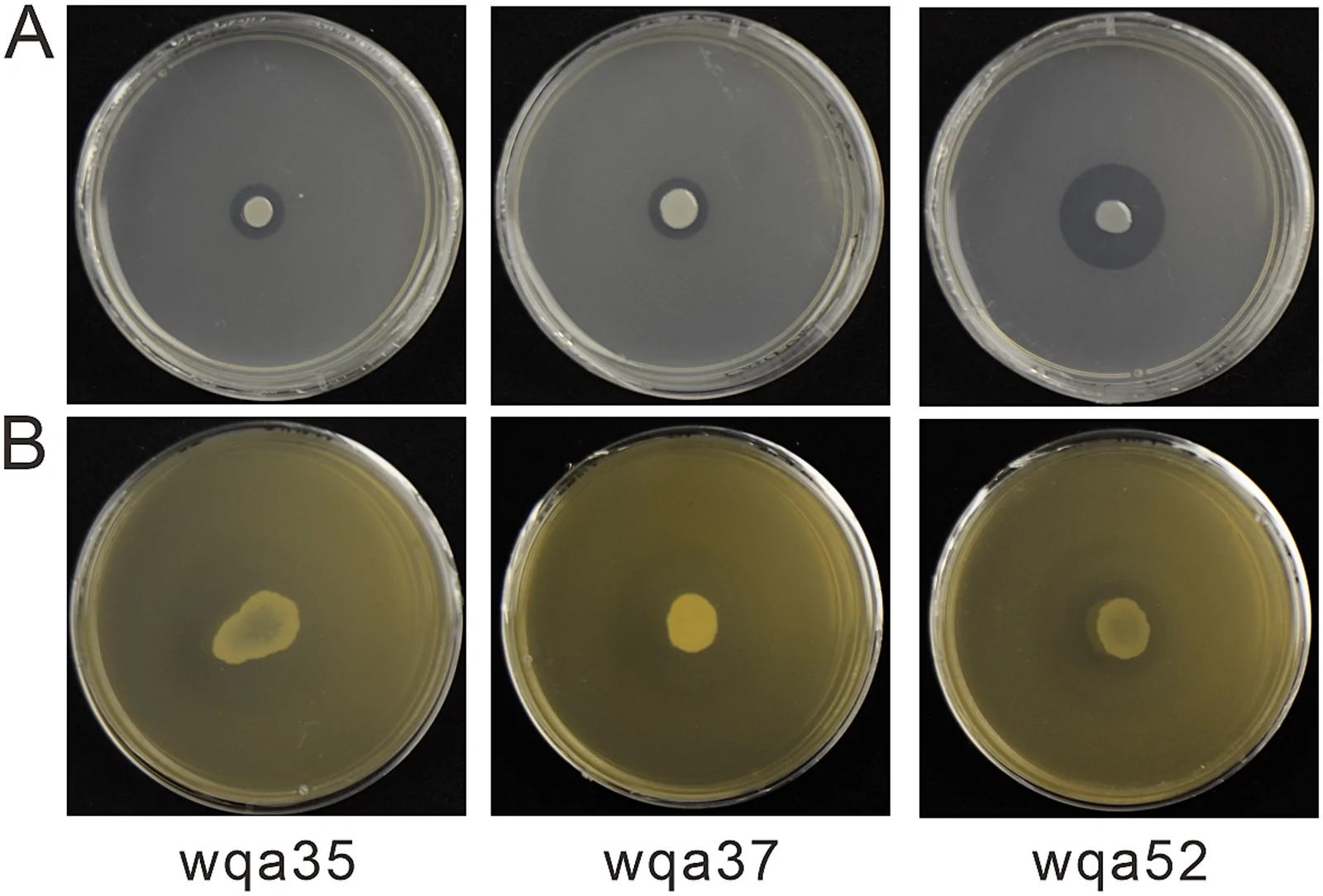
Phosphorus solubilization of bacterial strains isolated from Chinese fir root. (A) Inorganic phosphorus (Ca_3_(PO_4_)_2_) solubilization ability of bacterial strains. Photos were taken 5 days post inoculation. (B) Organic phosphorus solubilization ability of bacterial strains. Photos were taken 7 days post inoculation to Mongina organic phosphorus media.

**Table 1:** Species identification of the bacterial strains used in this study.

Strain	Color	Species identification
wqa34	Pale yellow	*Pseudomonas glycinae*
wqa35	White	*Burkholderia stagnalis*
wqa37	Darkened center, pale yellow around	*Burkholderia stagnalis*
wqa40	White	*Bacillus toyonensis*
wqa41	White	*Bacillus toyonensis*
wqa52	Dark yellow center, pale edge	*Paraburkholderia caffeinilytica*

To determine whether antagonistic interactions occurred among the six selected endophytic bacterial strains, all pairwise combinations were co-inoculated on LB agar, with the two inoculation points placed approximately one-quarter of the plate distance apart. Three biological replicates were included, and self-inoculation controls were set for each strain. Plates were photographed on day 5 after inoculation. Compared with the corresponding controls, colonies in the paired-inoculation treatments exhibited normal growth, indicating that no antagonism was detected among the six strains.

### Application of SynCom-P6 improve soil available phosphate content

To evaluate whether the synthetic community assembled from the six phosphorus-solubilizing endophytic bacteria (SynCom-P6) could increase soil available phosphorus, each strain was first cultured by shaking and then combined to form the synthetic community. SynCom-P6 was applied to test tubes containing equal volumes of soil and incubated in a growth chamber. Soil available phosphorus was measured on days 0, 7, 14 and 21, with three replicates per time point and a corresponding control group. Available phosphorus was quantified using both a spectrophotometer and a microplate reader, and the microplate reader results were summarized as boxplots (Figure 2). The boxplots showed that, from day 0 to day 7, soil available phosphorus in the treatment group decreased and was lower than that in the control. From day 7 to day 21, available phosphorus in the treatment group increased and significantly exceeded the control at day 14. These results suggested that, following inoculation, the synthetic community initially consumed part of the available phosphorus, but subsequently mobilized insoluble or unavailable soil phosphorus into plant-available forms that can be readily taken up by roots, thereby increasing soil available phosphorus.

**Figure 2:**
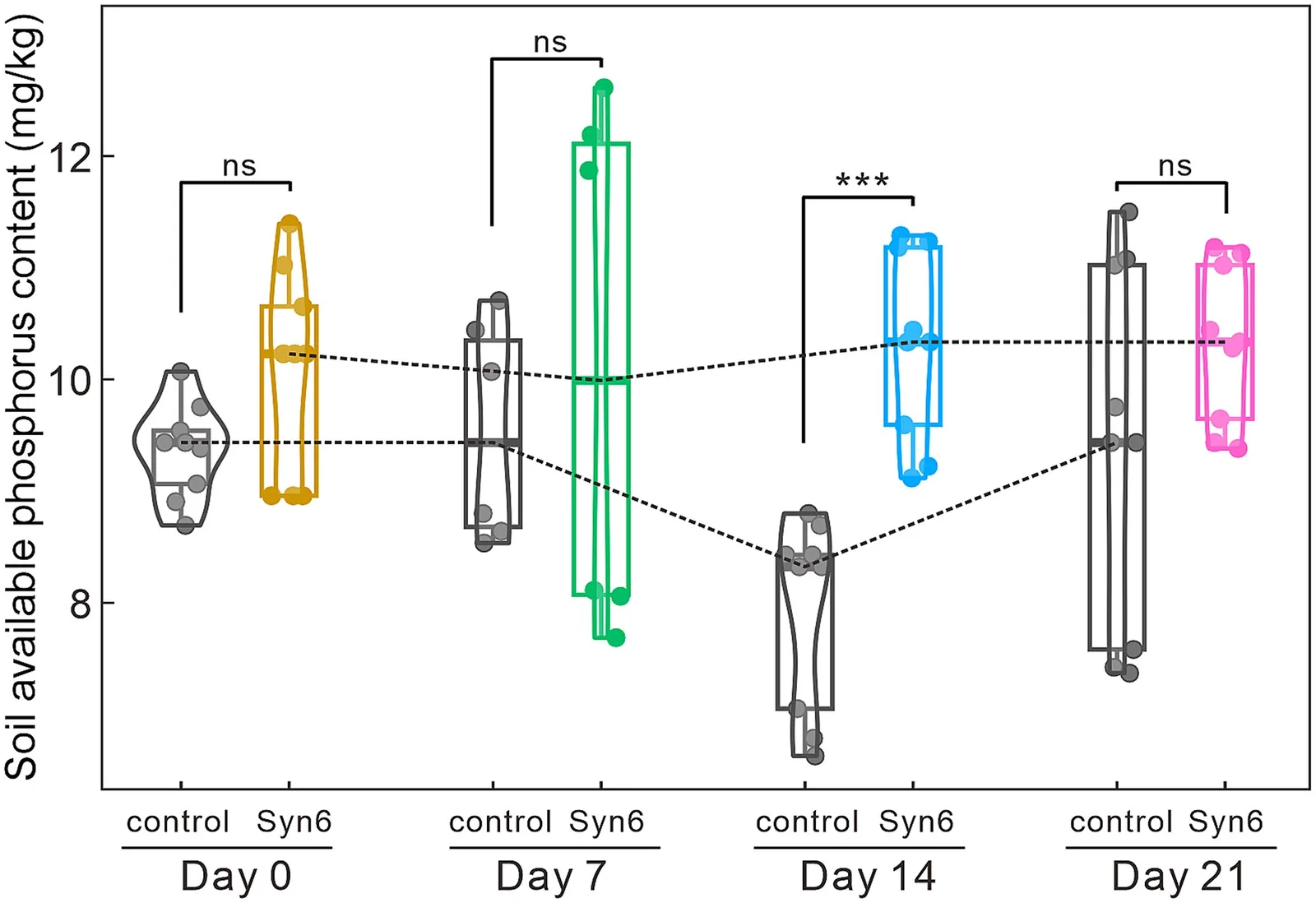
Soil available phosphorus content after the application of synthetic microbial community. The soil available phosphorus content was measured at day 0, day 7, day 14 and day 21 post addition of SynCom-P6. T-test was conducted to measure the difference between groups. Data were plotted using R package ggplot2. SynCom-P6: synthetic microbial community with six strains; control: control without microbes. ns: no significant difference, ^***^P < 0.001.

### Application of SynCom-P6 accelerate the growth of Chinese fir seedlings

Representative images of Chinese fir seedlings (Figure 3A) showed clear differences in root morphology between the SynCom-P6 treatment and the control group. Compared with the control, seedlings inoculated with SynCom-P6 exhibited additional developed root systems, characterized by markedly root length and root branching. Quantitative measurements further supported these morphological observations (Figure 3B). Seedling height did not differ between the SynCom-P6 treatment and the control. In contrast, root length was significantly increased under SynCom-P6 treatment (*P* < 0.01), indicating enhanced root growth.

**Figure 3:**
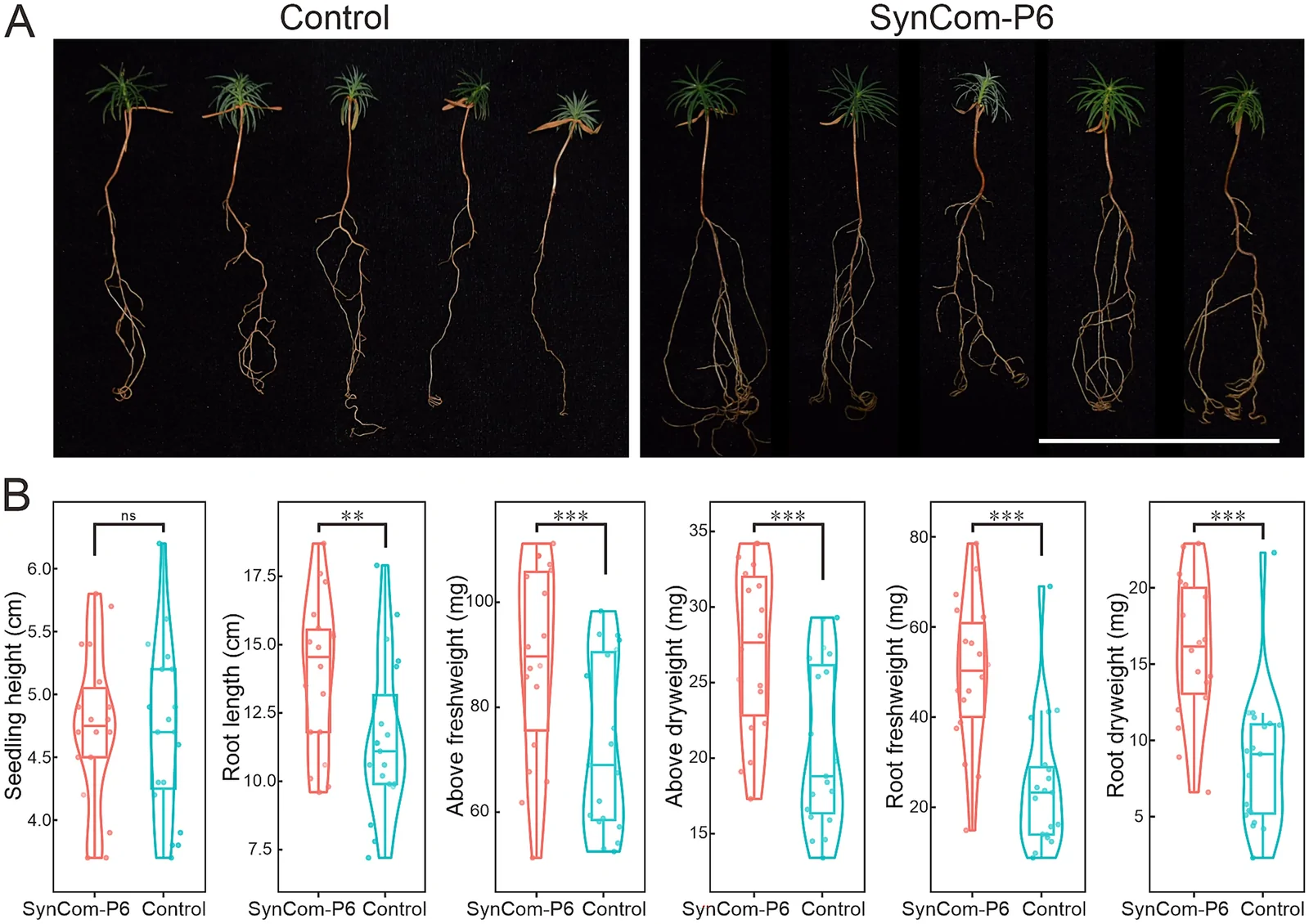
Root development promotion of Chinese fir seedlings treated by SynCom-P6. (A) Photos of Chinese fir seedlings treated with SynCom-P6 and control (10 mM MgCl_2_). Seedlings were treated with SynCom-P6 (OD = 0.2, 5 mL pot^−1^) or 10 mM MgCl_2_ (5 mL pot^−1^) at 4 weeks old. Photos were taken 5 months post treatment, bar = 10 cm. (B) Measurement of growth status of Chinese fir seedlings. ns: no significant difference, ^**^P < 0.01, ^***^P < 0.001.

Biomass analysis indicated that SynCom-P6 significantly affected both aboveground and belowground tissues. Compared with the control, both shoot fresh weight and shoot dry weight were significantly higher in the SynCom-P6 group (*P* < 0.001), suggesting increased aboveground biomass accumulation. Notably, the stimulatory effect of SynCom-P6 was more pronounced in roots. Both root fresh weight and root dry weight were significantly increased in the SynCom-P6 treatment relative to the control (*P* < 0.001), indicating a strong enhancement of root biomass accumulation. Overall, these results demonstrated that inoculation with the synthetic bacterial community SynCom-P6 significantly promoted root elongation and biomass accumulation in Chinese fir seedlings, and exerted a stronger effect on belowground growth at this developmental stage.

### SynCom-P6 changes rhizosphere microbial community with higher abundance of beneficial microbes

To evaluate the effect of SynCom-P6 inoculation on soil bacterial community structure, community composition was systematically assessed based on the absolute-quantification 16S amplicon dataset (Figure 4). The results showed that SynCom-P6-treated samples and controls shared a large number of common features, while each group also contained a certain number of unique features. This suggests that the two treatments retained conserved community components but also exhibited treatment-specific differences.

**Figure 4:**
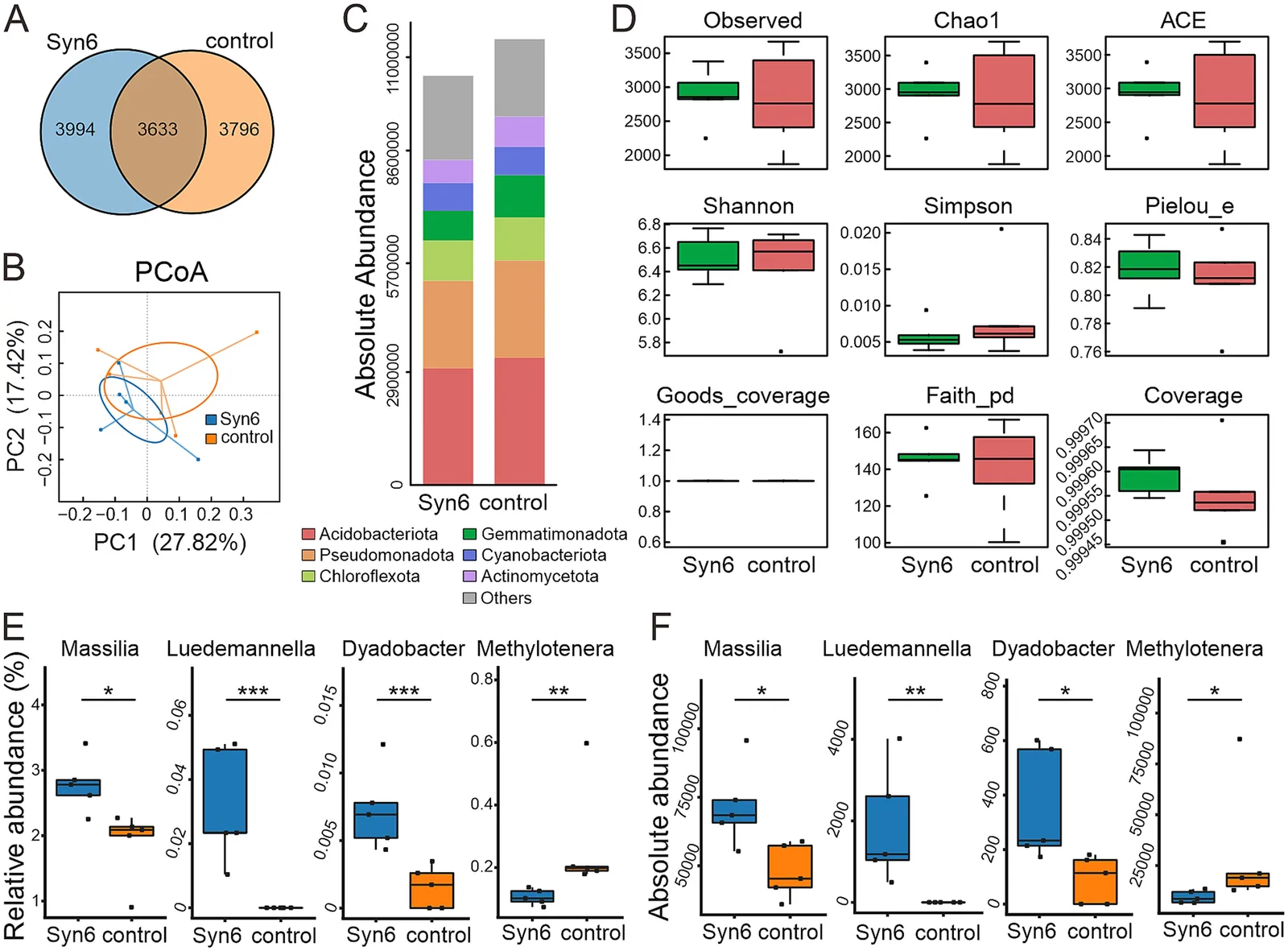
Absolute 16S Amplicon analysis for the rhizosphere microbial community. (A) Venn diagram showing shared and unique features between SynCom-P6 and control groups. (B) Bray–Curtis-based principal coordinate analysis (PCoA) of bacterial community structure from the absolute 16S dataset. (C) Absolute abundances of dominant bacterial phyla. (D) Wilcoxon test of alpha-diversity indices, including observed, Chao1, ACE, Shannon, Simpson, Pielou_e, Faith_pd and coverage. (E) Relative abundances of representative enriched genera. (F) Absolute abundances of representative enriched genera. Asterisks indicate significant differences between groups. Comparison between two groups was analyzed with t-test in R (^*^P < 0.05; ^**^P < 0.01; ^***^P < 0.001).

Bray–Curtis distance-based principal coordinate analysis (PCoA) further revealed a separation in community structure between the SynCom-P6 treatment and the control (Figure 4B). Specifically, the first principal coordinate (PC1) explained 27.82% of the variation and the second principal coordinate (PC2) explained 17.42%, together accounting for 45.24% of the total community variation. Samples in the SynCom-P6 group clustered more tightly in the ordination space, whereas control samples were more dispersed, indicating that SynCom-P6 inoculation significantly altered the overall community structure and increased community consistency within the treatment group.

At the phylum level, absolute abundance–based community profiling showed that the dominant taxa were broadly similar between SynCom-P6 and the control, although differences in absolute abundance were observed (Figure 4C). Comparisons of alpha-diversity indices derived from absolute abundance indicated that richness metrics (Observed, Chao1 and ACE), diversity indices (Shannon and Simpson), evenness (Pielou_e), phylogenetic diversity (Faith_pd) and sequencing coverage (Goods_coverage) did not differ significantly between the two treatments (Figure 4D). These results suggested that SynCom-P6 inoculation did not significantly affect overall bacterial richness or diversity.

In contrast, SynCom-P6 inoculation had a stronger impact on bacterial community composition at the genus level. Analyses of both relative and absolute abundance showed that *Massilia*, *Luedemannella* and *Dyadobacter* were significantly enriched in the SynCom-P6 treatment, whereas their abundances remained low in the control (Figure 4E and F). Overall, these findings indicate that SynCom-P6 reshaped the bacterial community primarily by promoting the enrichment of specific genera, rather than by inducing broad changes in overall alpha diversity.

To further explore the integrated effects of SynCom-P6 inoculation, both the absolute-quantification 16S amplicon and the metagenomic sequencing were conducted. Bray–Curtis distance-based principal coordinate’s analysis (PCoA) showed a clear separation in community structure between the SynCom-P6 treatment and the control (Figure 5A). Specifically, the first principal coordinate (PC1) explained 56.37% of the variation and the second principal coordinate (PC2) explained 14.71%, together accounting for 71.08% of the total community variation. These results indicated that SynCom-P6 inoculation exerted a strong effect on bacterial community structure. At the genus level, the Wilcoxon rank-sum test identified *Massilia*, *Desmonostoc*, *Nostoc* and *Planifilum* as significantly enriched in the SynCom-P6 treatment (Figure 5B), with absolute abundances consistently higher than those in the control.

**Figure 5:**
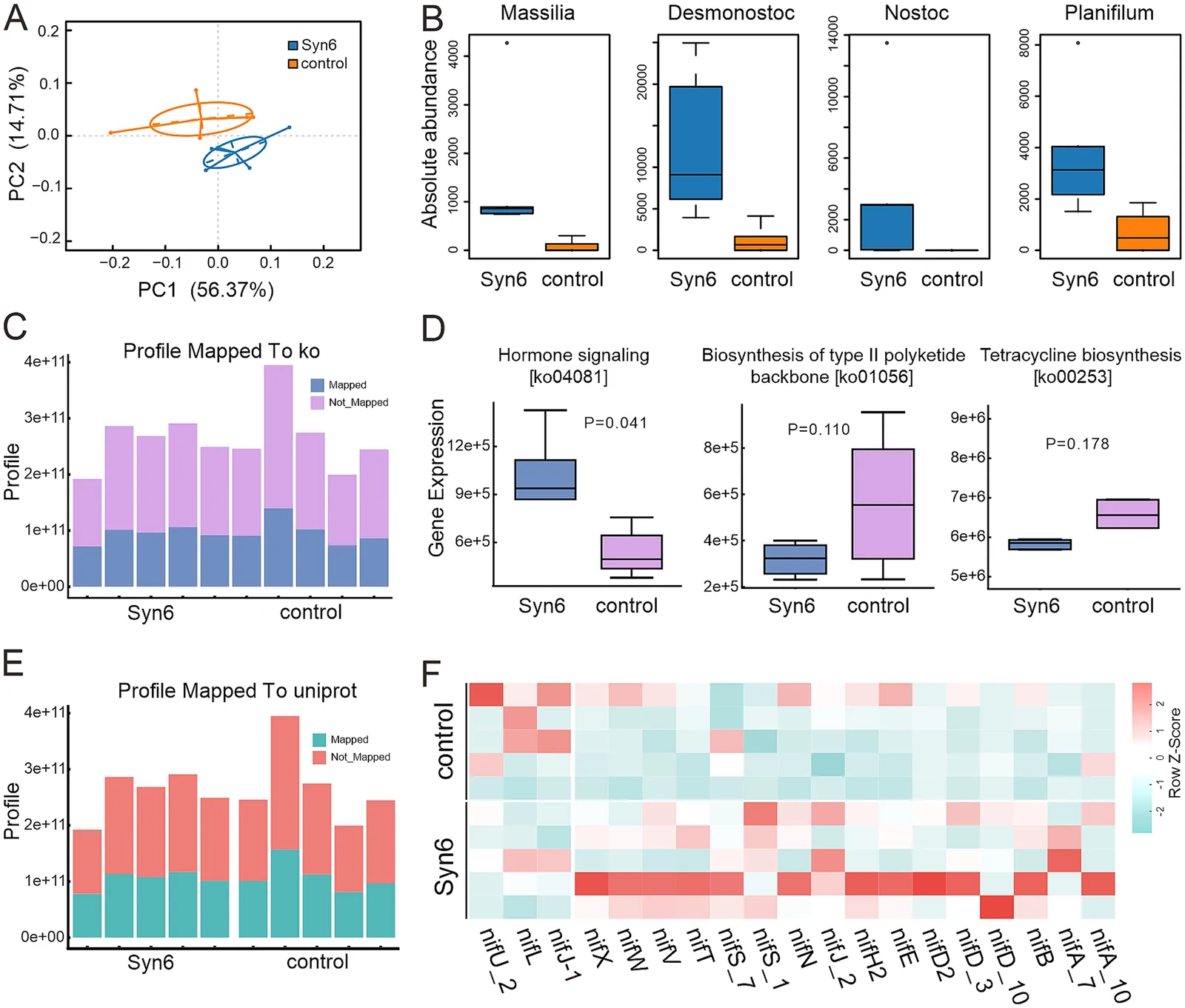
Metagenomic analysis for the rhizosphere microbial community. (A) Bray–Curtis-based principal coordinate analysis (PCoA) of microbial community structure from the absolute metagenomic dataset. (B) Wilcoxon analysis of absolute abundances of representative enriched genera, including *Massilia*, *Desmonostoc*, *Nostoc* and *Planifilum*. (C) Proportions of reads mapped and unmapped to KEGG Orthology (KO). (D) Absolute abundances of representative KO pathways, including hormone signaling [ko04081], biosynthesis of type II polyketide backbone [ko01056] and tetracycline biosynthesis [ko00253]. (E) Proportions of reads mapped and unmapped to UniProt. (F) Heatmap of nitrogen fixation-related genes, shown as row Z-scores.

Metagenomic functional profiling provided complementary functional evidence supporting the above community shifts. KEGG Orthology (KO) annotation showed that a fraction of sequences across all samples could be successfully mapped to KO, whereas a substantial proportion remained unannotated (Figure 5C). This result supports subsequent KO-based pathway comparisons while also highlighting limitations of current reference databases for soil metagenome annotation. Comparative analyses of representative KO pathways revealed that the hormone signaling pathway (ko04081) differed significantly between SynCom-P6 and the control (*P* = 0.041), and genes associated with this pathway were more abundant in the SynCom-P6 treatment. In contrast, type II polyketide backbone biosynthesis (ko01056) and tetracycline biosynthesis (ko00253) did not show statistically significant differences between treatments (*P* = 0.110 and 0.178, respectively) (Figure 5D).

UniProt-based functional annotation showed a similar pattern: while a proportion of functional genes could be annotated, a considerable fraction remained unannotated in all samples (Figure 5E). Heatmap analysis of nitrogen fixation–related genes further indicated that multiple *nif* genes were consistently enriched in the SynCom-P6 treatment, whereas their abundances were relatively low in the control (Figure 5F), suggesting a close association between SynCom-P6 inoculation and shifts in nitrogen fixation–related functional genes. Overall, the results demonstrated that SynCom-P6 inoculation not only significantly reshaped soil bacterial community structure, but was also accompanied by systematic changes in metagenomic functional profiles, including significant KO pathway differences and enrichment of nitrogen fixation–associated genes, highlighting the integrated influence of SynCom-P6 on the soil microbiome.

## Discussion

### Screening and application of phosphorus-solubilizing endophytic bacteria

In this study, screening of endophytic isolates revealed substantial differences among strains in their ability to solubilize inorganic phosphate. Previous studies have shown that phosphorus-solubilizing microorganisms can participate in inorganic phosphorus mobilization through multiple mechanisms, including organic acid secretion, chelation of metal ions and proton efflux. These mechanisms vary markedly across microbial taxa, which are consistent with observations reported in other plant systems such as maize, peanut and *Erythrina* spp. (Oteino et al. 2015, Berza et al. 2022, Santoyo et al. 2025). Recent studies further indicate that phosphate-solubilizing bacteria differ not only in solubilization efficiency but also in the physiological and molecular mechanisms underlying phosphorus mobilization. This may explain the strong variation observed among isolates in the present study and further justifies strain-level screening prior to SynCom-P6 assembly (Pan and Cai 2023). In addition, several isolates exhibited other plant growth–promoting traits, including indole-3-acetic acid production, phosphatase activity and siderophore synthesis (De Zutter et al. 2022), highlighting the functional diversity of endophytic phosphorus solubilizing bacteria. Together, these findings emphasize that selecting endophytes with strong and multifunctional phosphorus-solubilizing capacity is a prerequisite for constructing synthetic microbial communities. However, it should be noted that the specific physiological and biochemical mechanisms were not directly quantified in the present study (Santoyo et al. 2025). The endophytic origin of these strains may be particularly relevant, as endophytes are generally more closely associated with host physiology and may therefore exert more direct effects on plant growth and nutrient acquisition. This is consistent with previous reports showing that endophytic phosphate-solubilizing bacteria can directly promote plant growth after inoculation (Oteino et al. 2015, Semenzato and Fani 2024).

Our experimental results further demonstrated that the synthetic community significantly increased soil available phosphorus, and Chinese fir seedlings inoculated with this community showed improved morphological and physiological performance compared with normally grown seedlings. It has also been documented that phosphorus-solubilizing bacteria can promote plant growth and development, and increase leaf and root-shoot biomass (Xue et al. 2019). In a plant growth-promotion assay, the salt-alkali tolerant phosphorus-solubilizing strain *Bacillus* sp. DYS211 enhanced seed germination, with a promotion rate of 8.33% under high-salinity stress (Wang et al. 2023). Moreover, *Pseudomonas moraviensis*, *Bacillus safensis* and *Falsibacillus pallidus* have been reported to increase wheat yield by 14.42% while improving soil microbial biomass and activity (Wang et al. 2022). In maize, application of the phosphorus-solubilizing strains *Pantoea* sp., *Burkholderia cepacian* and *Acinetobacter baumannii* significantly altered rhizosphere microbial community structure and increased the relative abundance of Proteobacteria by 16.07–69.10% compared with the control (Luo et al. 2024). In addition, a synthetic phosphorus-solubilizing consortium was shown to promote tomato growth (Bakki et al. 2024). Collectively, these results support the potential application value of endophytic phosphorus-solubilizing bacteria in improving Chinese fir growth. Recent evidence further indicates that endophytic phosphate-solubilizing bacteria can enhance seedling growth and nutrient acquisition under specific soil conditions, supporting the broader relevance of endophytic PSB beyond crop systems and reinforcing their potential application in Chinese fir (Zhang et al. 2025).

### Construction of an endophytic SynCom-P6 in Chinese fir and its beneficial functions

After screening highly efficient phosphorus-solubilizing strains, we constructed a synthetic endophytic bacterial community to fully leverage functional complementarity and potential synergistic interactions among its members. Previous studies have shown that co-inoculation with endophytic and rhizosphere phosphorus-solubilizing bacteria outperforms single-strain inoculation in phosphate solubilization, root development and biomass accumulation (Emami et al. 2020, Luo et al. 2024). In addition, root exudates can enhance the phosphate-solubilizing activity of symbiotic bacteria, thereby further strengthening community-level functions (Pantigoso et al. 2023). Recent work has shown that phosphate-solubilizing function can be enhanced at the community level through artificial selection, supporting the view that phosphorus mobilization may emerge from collective community organization rather than from individual strains alone (Faller et al. 2024). In our experiments, application of the constructed SynCom-P6 to Chinese fir seedlings resulted in enhanced root elongation, increased root fresh and dry weights and increased aboveground biomass. These responses suggest that synergistic interactions among the selected endophytes improved nutrient acquisition and promoted plant growth. This observation is consistent with previous work showing that synthetic phosphorus-solubilizing consortia can reshape microbial community composition and enhance phosphorus–use efficiency (De Oliveira et al. 2025).

We also observed an increase in soil available phosphorus following SynCom-P6 inoculation. This effect is closely associated with the mechanisms by which the selected phosphorus-solubilizing strains mobilize different forms of inorganic phosphate. It has been reported that phosphate-solubilizing bacteria commonly employ three major strategies, including the release of organic acids, protons and siderophores; secretion of extracellular enzymes; and degradation of substrates through mineralization processes (Rawat et al. 2021). Our DNA-based species identification further confirmed the diversity of the selected phosphorus-solubilizing strains and their potential functional attributes. Numerous fungi and bacteria have been reported to solubilize elemental phosphate from insoluble phosphate sources to promote plant growth, including *Penicillium*, *Aspergillus* and *Pseudomonas* spp. (Sarker et al. 2014, Li et al. 2016). Moreover, isolated phosphorus-solubilizing bacteria can produce plant hormones such as auxin, gibberellin and cytokinin that promote plant growth (Panhwar et al. 2012, Rawat et al. 2021), as well as abscisic acid (ABA), which can enhance plant tolerance to abiotic stress (Khan et al. 2014). These findings help deepen our understanding of the roles of these microbes in soil phosphorus transformation. Such diversity may represent a key factor underlying the adaptability and stability of the synthetic community in complex soil environments. Studies on microbial interactions further suggest that different strains within a community can form mutually supportive relationships, thereby enhancing the overall phosphorus-solubilizing effect. Recent evidence further suggests that SynCom-P6 stability depends strongly on metabolic interactions and resource-use complementarity among members. Thus, in addition to the absence of antagonism, complementary resource use may also have contributed to the beneficial effects of SynCom-P6 (Wang et al. 2025).

These findings further suggested that applying artificial phosphorus-solubilizing bacteria may reduce soil dependence on phosphate fertilizer inputs, thereby supporting a transition toward inoculation with artificially cultivated endophytic communities. Long-term and excessive use of conventional phosphate fertilizers often leads to soil acidification and phosphorus loss, which in turn contributes to global groundwater and surface-water pollution (Craswell 2021), as well as heavy metal contamination (Mehmood et al. 2009, Niño-Savala et al. 2019). In this context, we propose that replacing some chemical phosphate fertilizers with endophytic phosphorus-solubilizing consortia may help slow the progression of soil acidification over long-term management, thereby improving soil quality and reducing the risk of phosphorus loss. Such an eco-friendly management strategy may not only promote Chinese fir growth but also provide benefits for environmental protection (Xu et al. 2025).

Beyond functional screening, the construction of an effective synthetic microbial community also requires consideration of multiple ecological and biological factors (Jing et al. 2024). Compared with single-strain inoculation, synthetic communities often exhibit superior performance, suggesting functional complementarity among members with distinct metabolic roles (Niu et al. 2017). Interactions among community members, including cooperation and competition, can further shape community stability and functional expression; for instance, antagonism may suppress beneficial effects (Weiland-Bräuer 2021). Moreover, niche differentiation can reduce direct resource competition and facilitate stable coexistence among community members. The stable coexistence of multiple taxa observed in synthetic communities under different environmental conditions highlights the importance of resource partitioning in reducing competition and maintaining community persistence (Goldford et al. 2018). Taken together, these considerations indicate that rational design of endophytic SynComs should integrate both functional and ecological dimensions, rather than relying solely on single-trait screening.

### Limitations and future research directions

Although this study successfully constructed an endophytic synthetic community with phosphorus-solubilizing capacity in Chinese fir and validated its promoting effects on soil available phosphorus and root development in seedlings, several limitations remain. Firstly, the experiments were mainly conducted under controlled conditions and therefore may not fully reflect the complexity and heterogeneity of natural forest soils. Thus, the colonization capacity and functional stability of SynCom-P6 under field conditions require further verification. The pronounced root response observed here warrants further mechanistic investigation. Recent studies indicate that root plasticity is strongly influenced by microbial partners, suggesting that the increase in root length and root biomass after SynCom-P6 inoculation may reflect broader microbiome-mediated regulation rather than a phosphorus effect alone (Dini-Andreote et al. 2025). Secondly, although we documented an increase in soil available phosphorus and improvements in root phenotypes, the mechanisms by which the synthetic community promotes phosphate solubilization and root development remain largely descriptive. Specifically, the interactions among community members, the expression of key enzyme-encoding genes, and signal communication with host root exudates were not systematically investigated, despite their potential direct influence on phosphorus transformation efficiency and plant responses. In addition, this study did not consider potential synergistic effects with other microorganisms; for example, co-application of arbuscular mycorrhizal fungi (AMF) and phosphorus-solubilizing bacteria has been reported to significantly enhance plant phosphorus acquisition (Saxena et al. 2013).

Future research should aim to address these gaps. Most importantly, validation of the synthetic endophytic community should be extended to natural forest environments, covering diverse soil types, seasonal variation and different soil nutrient conditions, which would provide a deeper understanding of the robustness and adaptability of this community across ecological contexts. Furthermore, we should evaluate the practical performance of this SynCom-P6 under large-scale forestry applications. This includes conducting long-term field trials and monitoring tree growth, phosphorus dynamics, and microbial community succession, thereby assessing its adaptability and functional persistence under different cultivation conditions. Moreover, multi-omics approaches (e.g. genomics and metabolomics) should be employed to elucidate how microbial interactions within SynCom-P6 and the expression of functional genes drive phosphate solubilization and root development (Ding et al. 2021). Given the well-documented synergistic effects between phosphorus-solubilizing bacteria and AMF, combined inoculation strategies should also be explored to further enhance nutrient acquisition efficiency (Zhang et al. 2018, Cozzolino et al. 2021). Overall, these research directions will provide a scientific basis for the sustainable management of Chinese fir plantations and offer valuable insights for nutrient management in other plant species. In conclusion, this study effectively demonstrated the potential of endophytic phosphorus-solubilizing bacteria to enhance Chinese fir growth and increase soil available phosphorus. Moreover, we proposed an innovative soil management strategy based on synthetic microbial communities. With continued technological advances, this strategy is expected to play an increasingly important role in the management and conservation of forest ecosystems.

## Data Availability

Metagenomic and 16S amplicon raw reads have been deposited into the NCBI Sequence Read Archive (SRA) database with accession number PRJNA1443062 (reviewer link: https://dataview.ncbi.nlm.nih.gov/object/PRJNA1443062?reviewer=ov2oja34eisruht4ts74jb20a5).
